# Minimally invasive protocols and quantification for microglia-mediated perineuronal net disassembly in mouse brain

**DOI:** 10.1016/j.xpro.2021.101012

**Published:** 2021-12-11

**Authors:** Alessandro Venturino, Sandra Siegert

**Affiliations:** 1 Institute of Science and Technology (IST) Austria, Am Campus 1, 3400 Klosterneuburg, Austria

**Keywords:** Bioinformatics, Immunology, Microscopy, Model Organisms, Neuroscience

## Abstract

Enzymatic digestion of the extracellular matrix with chondroitinase-ABC reinstates juvenile-like plasticity in the adult cortex as it also disassembles the perineuronal nets (PNNs). The disadvantage of the enzyme is that it must be applied intracerebrally and it degrades the ECM for several weeks. Here, we provide two minimally invasive and transient protocols for microglia-enabled PNN disassembly in mouse cortex: repeated treatment with ketamine-xylazine-acepromazine (KXA) anesthesia and 60-Hz light entrainment. We also discuss how to analyze PNNs within microglial endosomes-lysosomes.

For complete details on the use and execution of this protocol, please refer to Venturino et al. (2021).

## Highlights

• PNN disassembly with repeated ketamine-mediated anesthesia in mice
• 60 Hz light entrainment as an alternative approach
• Strategy to quantify density of PNN-coated cells
• Determine PNN material enriched within microglial lysosomes

## Graphical abstract

**Figure:**
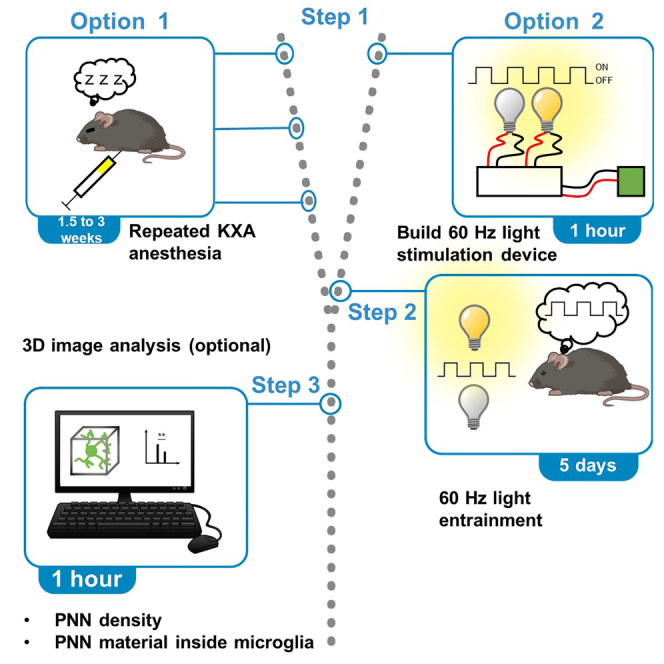

## Before you begin

We outline two protocols for microglia-enabled PNN disassembly in the primary visual cortex, which we have applied in C57BL/6J mice of both sexes at the age of 8–12 weeks.

### Protocol 1: KXA (ketamine-xylazine-acepromazine)

The first protocol (option 1) is based on repeated exposure to ketamine-mediated anesthesia. Ketamine is an anesthetic that amongst others blocks the NMDA receptor (Zorumski et al., 2016) and preferentially acts on parvalbumin-positive neurons (Behrens et al., 2007).

In our lab, we have applied this protocol with the following parameters: (1) C57BL/6J (8–12 weeks of age, both sexes), 1×, 2×, 3×, 6× KXA for primary visual cortex (V1) and primary somatosensory cortex (S1) in a repetition-dependent manner. We observed for both, V1 and S1, approximately 80% and up to 90% PNN loss with 3× and 6× KXA, respectively (Venturino et al., 2021); (2) C57BL/6N (8–12 weeks of age, both sexes), 3× KXA for V1 and S1. C57BL/6N mice showed 80% reduction of PNN density after 3× KXA treatment, in line with the result obtained with C57BL/6J mice (Venturino et al., 2021).

***Note:*** Common mouse models for labeling microglia like CX_3_CR_1_^CRE-ERT2^ or CX_3_CR_1_^GFP^ are knock-in models that will contain a CX_3_CR_1_ knock-out allele, when used as heterozygous animals, or CX_3_CR_1_ fully knocked out, when homozygous. This might alter the KXA-mediated microglia response as *e.g.* CX_3_CR_1_ is involved in synapse pruning and injury responses (Paolicelli et al., 2011; Freria et al., 2017)*.*

***Note:*** Our animal protocol has so far only covered the combination of ketamine-xylazine-acepromazine (KXA) but the procedure also works with 3× KX in the V1 (Venturino et al., 2021).

***Note:*** 20% of PNN recovers within 3 days after the last 3× KXA injection and reaches full coverage by 14 days for S1 and V1 of C57BL/6J mice (Venturino et al., 2021). The recovery kinetics may differ between the mouse model and strain background.

### Protocol 2: 60-Hz light entrainment

The second protocol (option 2) focuses on the visual stimulation protocol and uses 60 Hz light flickering entrainment. Here, the pulsed light mimics ketamine-induced gamma oscillation, which has been previously described in the mammalian brain (Akeju et al., 2016). We applied this protocol in C57BL/6J mice (8–12 weeks, both sexes), 2 h daily for 5 days for V1. We did not observe PNN loss when we applied the same paradigm with 8 or 40 Hz pulsed light compared to controls (treated with non-flickering light with the same light intensity). As a note, it has been shown that gamma oscillations induced by 40 Hz light stimulation can propagate to other brain regions such as hippocampus (CA1) (Iaccarino et al., 2016) and frontal cortex (Jones et al., 2019), where this stimulation paradigm triggers microglia-mediated engulfment of amyloid-β plaques in murine models of Alzheimer disease (Iaccarino et al., 2016; Martorell et al., 2019).

For both protocols, we identified microglia as the main cell type for promoting PNN degradation as described in Venturino et al. (2021). Both protocols failed to dismantle PNNs in microglia-depleted mice, where approximately 80% of the microglial cells were depleted with PLX 5622-enriched food administered 1.5 weeks prior and during both treatment regimens (Venturino et al., 2021). The condition for protocol 1 were C57BL/6J (8–12 weeks of age, both sexes), 3× KXA for V1 and S1) and protocol 2, C57BL/6J (8–12 weeks of age, both sexes), 2 h daily for 5 days in V1.

**CRITICAL:** Before starting with the experiments, it is important for the users to have the approval of the animal procedures from the ethical committee of their institute and all relevant authorities.

## Key resources table

**Table:** 

REAGENT or RESOURCE	SOURCE	IDENTIFIER
**Antibodies**		

Rat α-CD68 (1:250)	Bio-Rad AbD Serotec Limited	Cat#MCA1957, clone FA-11, Lot 1807, RRID:AB_322219
Goat α-Iba1 (1:200)	Abcam	Cat#ab5076, Lot FR3288145-1, RRID:AB_2224402
Rabbit a-Iba1 (1:750)	GeneTex	Cat#GTX100042, Lot 41556, RRID:AB_1240434

**Chemicals, peptides, and recombinant proteins**		

*Wisteria floribunda* lectin – fluorescein-labeled (1:200)	Szabo-Scandic	Cat#VECFL-1351, Lot ZE0611
*Wisteria floribunda* lectin – biotinylated (1:200)	Szabo-Scandic	Cat#VECB-1355, Lot ZE0424
Hoechst 33342 (1:10.000)	Thermo Fisher Scientific	Cat#H3570
Isoflurane	Zoetis	Cat#6089373
Ketamine	MSD Animal Health	Cat#A137A01
Xylazine	AniMedica	Cat#7630517
Acepromazine	VANA GmbH	Cat#18F211

**Experimental models: Organisms/strains**		

Mouse: C57BL/6J	The Jackson Laboratory	Cat#000664

**Software and algorithms**		

MATLAB R2019b	MathWorks	RRID:SCR_001622
Imaris	Bitplane Imaris	9.3.1
Arduino IDE for PC or Mac	https://www.arduino.cc/en/main/software	N/A

**Other**		

Arduino UNO	RS Components	Cat#769-7409
Breadboard	RS Components	Cat#102-9147
Transistor	RS Components	Cat#485-7721
200 Ω resistor	RS Components	Cat#174-3016
Jumper wires	RS Components	Cat#634-8651
White LED strip	RS Components	Cat#153-3638
Box 62 l with lid (black)	OBI	Cat#4969176
Soldering set	OBI	Cat#4162129
Insulating tape	OBI	Cat#7613235

## Materials and equipment

The stock solution of ketamine (100 mg/mL), xylazine (20 mg/mL) and acepromazine (10 mg/mL) are obtained from the company. The drugs are in saline solution in “ready-to-use” formulation. The drugs have to be stored between 2°C and 8°C. The expiration date indicated on the vial should not be exceeded.

## Step-by-step method details

### Option 1. PNN disassembly by repeated KXA-induced anesthesia

**Timing: 1–3 weeks**

Here, we describe first how to prepare a ready-to-use KXA solution, followed by performing the intraperitoneal injection, and finally we outline how to check the level of anesthesia and how to maintain the animals during the anesthetized state.

The drug dosages used for mice are: (1) ketamine 100 mg/kg; (2) xylazine 10 mg/kg; (3) acepromazine 3 mg/kg.

***Note:*** The effect of PNN loss also occurs with just ketamine and xylazine. On the other hand, acepromazine reduces the death rate and improves the surgical plane (Arras et al., 2001).

**CRITICAL:** Ketamine at this dosage must be combined with xylazine to prevent uncontrolled muscular contraction.

1. Prepare the ketamine-xylazine-acepromazine (KXA) solution.
  a. Combine 2 mL of ketamine, 1 mL of xylazine, and 0.6 mL of acepromazine in 3 mL of physiological saline solution containing 0.9% (w/v) NaCl.
  b. Vortex for 30 s.
  c. The KXA solution should look yellow without any precipitates and can be stored for up to 2 weeks at 4°C. Troubleshooting 1
2. Weigh the mice on a fine scale.
3. Calculate the injection dosage for each mouse. A mouse receives 33 μL/10 g weight e.g., a mouse weighing 30 g receives 99 μL of the KXA solution (Harkness and Wagner, 1995).

**CRITICAL:** The drug dosage must be adjusted for each mouse. Even if mice are the same sex or come from the same litter, there may be fluctuations in the body weight.

**Figure 1:**
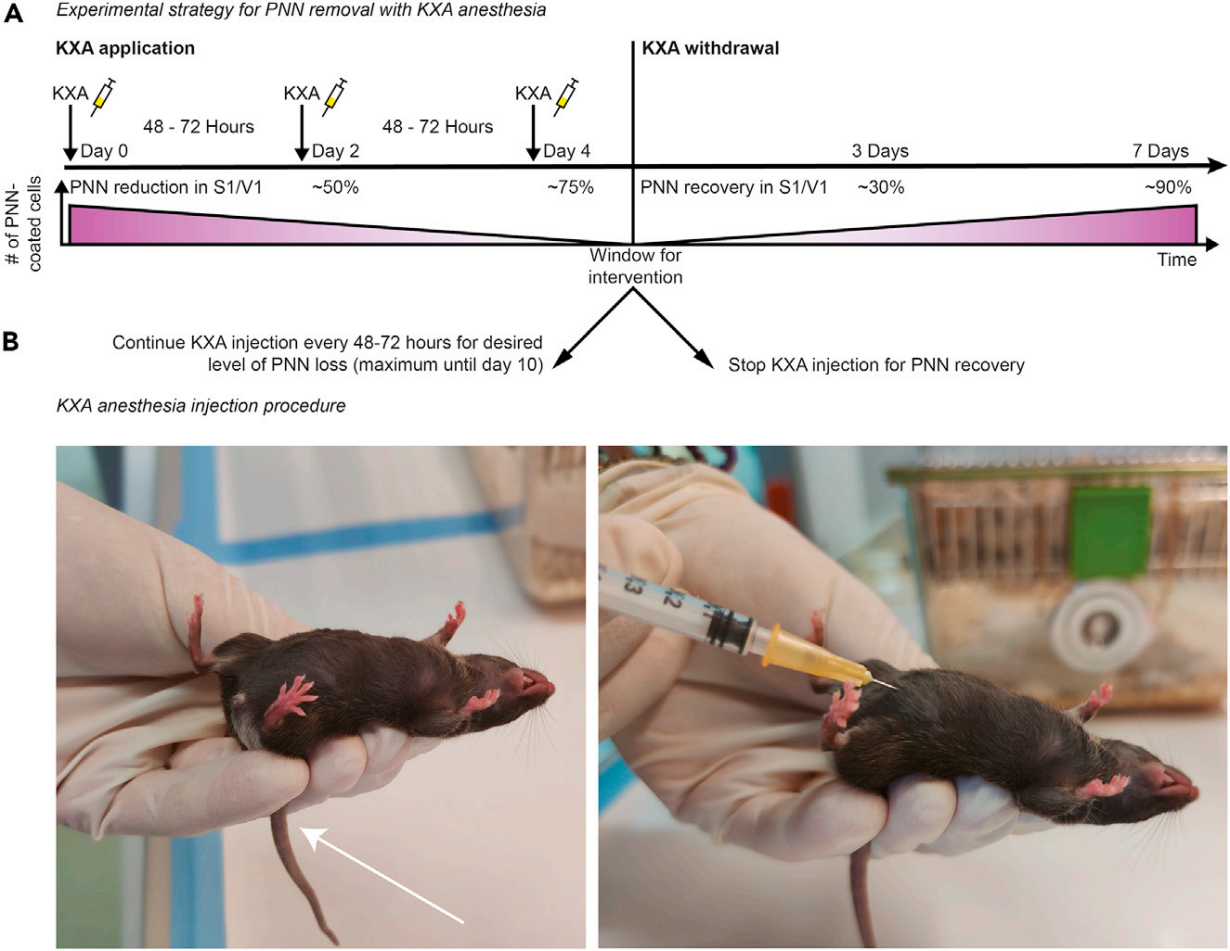
PNN disassembly by repeated KXA-induced anesthesia (A) Experimental timeline for repeated KXA application including the expected PNN loss and recovery rate after KXA exposure. KXA, ketamine-xylazine-acepromazine. PNN, perineuronal net. S1, primary somatosensory cortex. V1, primary visual cortex. (B) Mouse receiving injection. Left, restraining position for *intraperitoneal* (*i.p.*) injection. Arrow, position of the tail between the ring and the pinky of the operator. Right, the injection procedure, in which the needle is inserted with a 45° angle into the lower quadrant of the abdomen.

***Note:*** Tilting the mouse head down helps push the internal organs towards the head, which prevents damage from the injection.

4. Prepare a 1 mL syringe equipped with a 26G needle with the appropriate amount of KXA solution.

5. Inject intraperitoneally. A schematic of the repeated KXA treatment protocol can be found in Figure 1A)
  a. Grab the mouse at the neck and turn it around (Figure 1B).
  b. Insert the needle at 45° into the peritoneal cavity (Figure 1B).
    **CRITICAL:** Do not inject on the *linea alba*. This might cause lesions to the bladder.
    **CRITICAL:** Make sure that the solution does not leak out of the mouse.
    ***Note:*** After the injection, no solution should accumulate and be visible under the skin.
  c. Turn the mouse back, head down, and gently release it back into its home cage.

**CRITICAL:** The injection must be performed intraperitoneally with the needle inserted directly into the peritoneal cavity. In our experience and with the anesthetic dosage used in this protocol, misplacement will cause inefficient anesthesia induction. As a note, it has been shown that ketamine anesthesia can be achieved with subcutaneous administration in mice with double the concentration used here (Levin-Arama et al., 2016).

6. Monitor the state of anesthesia.
  a. After the injection, the mouse will undergo a hyperexcitation phase for the first 1–2 min marked by increased running activity. Then, the mouse will slowly become unconscious, and the anesthesia will begin. It might take 5–10 min to reach the induction of the surgical plane, which will last for approximately 40 min. Troubleshooting 2
  b. Apply eye ointment to prevent dehydration of the sclera.
  c. Confirm the anesthesia by checking the following parameters (Tsukamoto et al., 2015):
    i. Absence of the toe pinch reflex around 10 min after induction.
    ii. Decrease in respiratory frequency.
    iii. No responses to noxious stimuli.
    iv. Flaccid paralysis.
    v. Absence of whisker movement.

**CRITICAL:** To prevent hypothermia, the mouse should be kept at 37°C with a heating pad or a red lamp for the entire time (induction, deep anesthesia and recovery phase).

***Note:*** Regularly check temperature with a thermometer and adjust the heating device accordingly.

7. After complete recovery, return the mouse to the animal facility with food and water *ad libitum.*

***Note:*** If many animals are housed in the same cage, make sure that all animals are awake, as they otherwise become aggressive to the ones that are still waking up.

***Note:*** To avoid potential impact of circadian rhythm on brain activity (Hor et al., 2019), we always injected the animals at the same time (in our case 9 a.m.) and collected the tissue 4 h after the last treatment.

**Figure 2:**
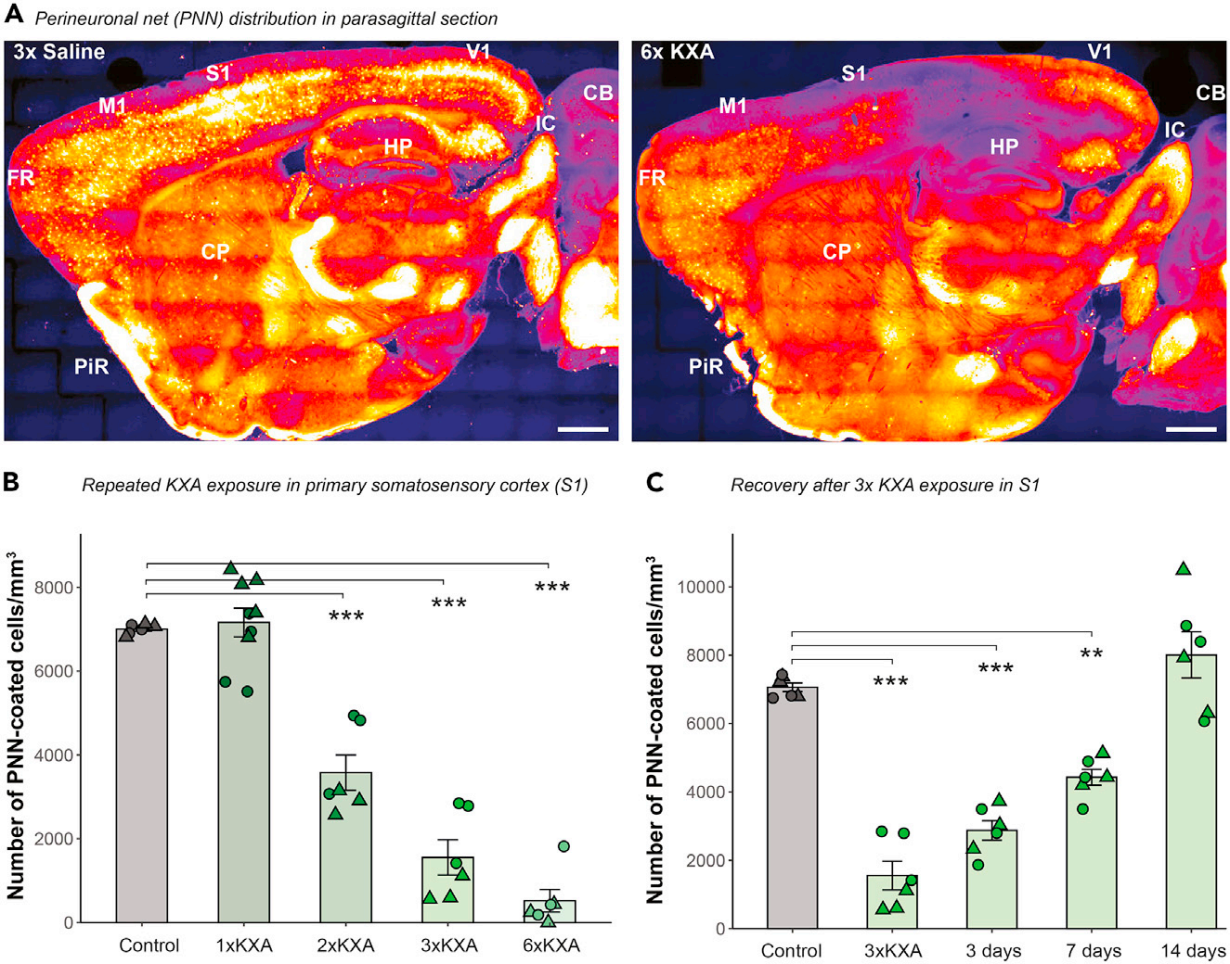
Expected results for PNN loss and recovery with repeated KXA-induced anesthesia (A) Wisteria floribunda agglutinin (WFA)-labeled parasagittal brain sections after 6× saline (left), and 6× KXA treatment (right). Scale bar: 1000 μm and 800 μm, respectively. Color intensity reflects PNN density with blue and white low and high density, respectively. CB, cerebellum. CP, caudato putamen. FR, frontal cortex. IC, inferior colliculus. M1, primary motor cortex. PIR, piriform cortex. S1, primary somatosensory cortex. V1, primary visual cortex. (B and C) PNN density in S1. Absolute number of PNN-coated cells ± SEM after repeated KXA treatment (B) and after 3× KXA withdrawal (C). Triangle, female. Circle, male. Linear regression model with selected post-hoc comparison. ∗∗p < 0.01. ∗∗∗p < 0.001, ^ns^p > 0.05. SEM: standard error of the mean.

***Note:*** We typically performed our analyses 4 h after the last injection.

***Note:*** 20% of PNN recovers within 3 days after the last 3× KXA injection and reaches full coverage by 14 days for S1 (Figure 2C) and V1 of C57BL/6J mice (Venturino et al., 2021).

***Note:*** PNN recovery kinetics may differ between the mouse model and strain background.

***Note:*** We observed a significant reduction in the number of PNNs in S1 and V1 after repeated KXA anesthesia (Figure 2A). The effect of this treatment may not be the same in all brain regions because ketamine does not act equally throughout the brain (Alda et al., 1994). For example, we found PNN removal in the hippocampus only after 6× KXA (Venturino et al., 2021).

8. Repeat steps 1–7 every 2–3 days at least two times (up to 6 times) to reach the desired level of reduction in the number of PNN-coated cells (Figure 1A). Expected results for 6× KXA treatment can be found in Figures 2A and 2B.

### Option 2. PNN disassembly by 60-Hz light entrainment

This section describes first how to build and program a 60 Hz light stimulation device, and then how to verify the stimulation frequency. Steps 9–17 outline the building of the hardware, steps 18–24 the software.

#### Part 1. Build light stimulation device for PNN disassembly with 60 Hz

**Timing: up to 1 h**

**Figure 3:**
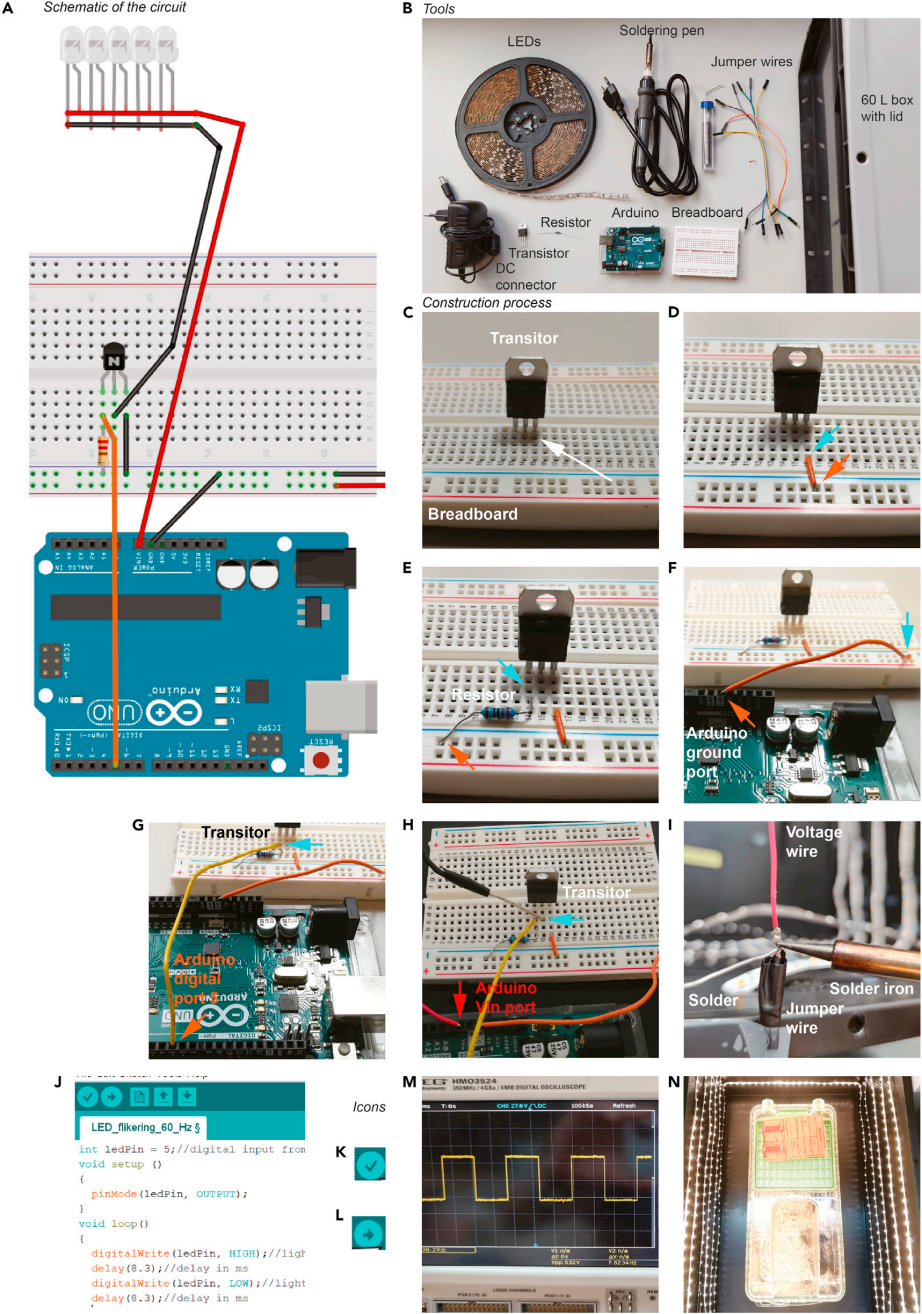
Construction of the light stimulation device (A) Schematic of the circuit designed with the software Fritzing. (B) Overview of the necessary tools. (C–I) Step-by-step constructions of building the electric circuit. (C) Transistor is inserted on the breadboard. White arrow, ground pin. (D) Electric connection built between the transistor ground (blue arrow) and the breadboard ground (orange arrow). (E) The resistor is connected with the left transistor pin (blue arrow) to the breadboard ground (orange arrow). (F) The orange cable connects the Arduino ground port (orange arrow) to the breadboard ground pin (blue arrow). (G) The yellow cable connects the Arduino digital port 5 (orange arrow) to the left transistor pin (blue arrow). (H) The red LED voltage cable is connected to the Arduino Vin port (red arrow). The grey jumper wire to the transistor exit pin (blue arrow). (I) Soldering the red voltage wire from the LED strip to a jumper wire. (J) Screenshot of Arduino IDE software user-interface for the board programming. (K and L) Code “check” and “upload” Arduino IDE icons, respectively. (M) Oscilloscope reading of the Arduino digital port 5 output showing 60 Hz square wave function. (N) Adjust a mouse cage to the center of the stimulation box for equal illumination.

An overview of the circuit and the components needed for the assembly of the light stimulation device can be found in Figures 3A and 3B.

9. Stick the LED strip horizontally along the inner walls of a black plastic box (60 × 40 × 32 cm, 62 l volume). The LED strip goes round the box in 6 layers with 4 cm space in between.

***Note:*** Add the LED stripes in a way from top to bottom to allow equal illumination.

10. The circuit is built as outlined in Figure 3A.
11. Take the breadboard and insert the transistor. The ground pin of the transistor is on the right (Figure 3C).

***Note:*** The ground pin of the transistor is often indicated on the chip with the letter “G”.

12. Connect the ground of the breadboard (blue symbol “−”) with the ground of the transistor (Figure 3D).
13. Put a 200 Ω resistor between the ground of the breadboard and the left pin of the transistor (Figure 3E).

***Note:*** A resistor is critical for the functionality of the transistor.

14. Connect one ground port of the Arduino board (indicated with the letters GND) to the ground of the breadboard (blue symbol “−”) (Figure 3F).
15. Connect the digital port 5 of the Arduino board to the left pin of the transistor (Figure 3G).

***Note:*** The resistor should be in front of the cable that goes to the left pin of the transistor.

***Note:*** The number of the Arduino digital port depends on the programming code. We used digital port 5 in our code to send the signal to the LEDs.

16. Connect the voltage wires from the LED strip as follows:
  a. the red cable is connected to the Arduino board at the Vin (voltage in) port, and
  b. the black cable to the middle pin of the transistor (Figure 3H).

***Note:*** If the cable is a fine-stranded conductor, solder a jumper wire on the cable to allow a stable connection with the transistor on the breadboard (Figure 3I).

**CRITICAL:** Insulate the solder joint with electrical tape.

17. The system is connected to a 12 V power supply via the jack plug of the Arduino.
18. Download and install the Arduino IDE for PC or Mac from this link: https://www.arduino.cc/en/main/software
19. Connect the Arduino board to the laptop with a USB cable type A/B.
20. Open the Arduino IDE (Figure 3J) and select the Arduino board in *Tools > Board > Arduino/Genuino UNO.*

***Note:*** Ensure that the correct Arduino board is selected.

21. Copy and paste the following codes:
  a. for the 60 Hz flickering in the Arduino IDE text editor:
    int ledPin = 5;//digital input from the board
    void setup ()
    {
     pinMode(ledPin, OUTPUT);
    }
    void loop()
    {
     digitalWrite(ledPin, HIGH);//light on, no fading
     delay(8.3);//delay in ms
     digitalWrite(ledPin, LOW);//light off, no fading
     delay(8.3);//delay in ms
    }
  b. for the control with the same light intensity without 60 Hz flickering.

int ledPin = 5;//digital input from the board

void setup ()

{

 pinMode(ledPin, OUTPUT);

}

void loop()

{

 digitalWrite(ledPin, HIGH);//keep light on

}

***Note:*** In this script, we use digital port 5 to send the signal to the LEDs. If another digital port is used, the script has to be modified accordingly.

22. Verify that the script is functional by clicking on the “check” icon (Figure 3K).

***Note:*** If the script doesn’t work the Arduino IDE will show a red error message reporting in which line of the code the problem is. Troubleshooting 3

23. Select the output port for uploading the code in *Tools > Port.* In our example, the port is COM5.

***Note:*** Ensure that the port number corresponds to your Arduino.

24. Upload the code from step 21 by clicking on the “upload” icon (Figure 3L).

***Note:*** The Arduino board memorizes previously uploaded code. Each time the board is powered up it will execute the code automatically.

25. Confirm with an oscilloscope that the frequency (60 Hz) and the shape (square wave) of the stimulation is correct (Figure 3M).
26. Take a mouse cage, adjust it to the center of the stimulation box, and mark the position with a tape (Figure 3N).

#### Part 2. PNN disassembly with 60 Hz light entrainment

**Timing: 5 days**

**Figure 4:**
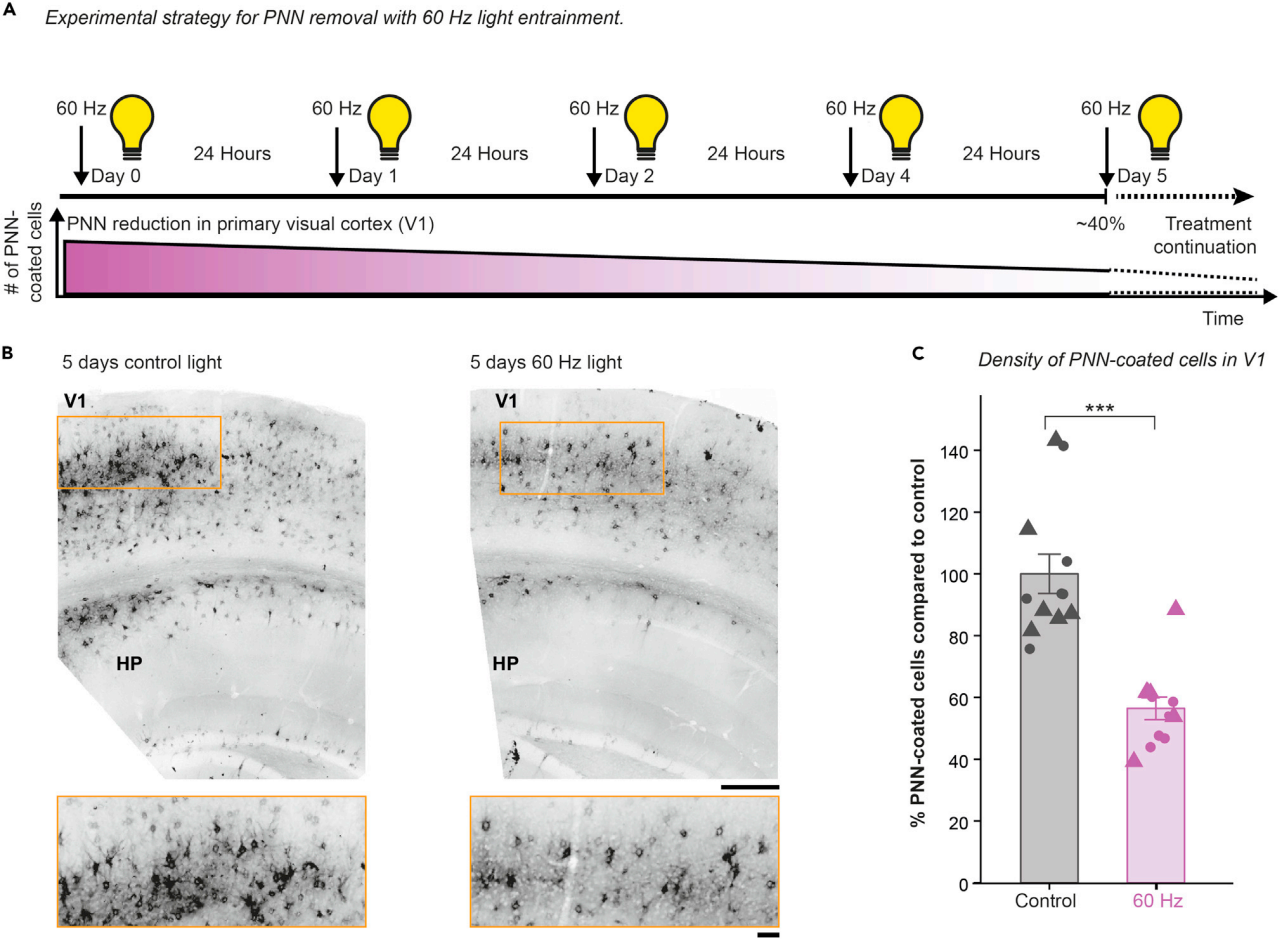
PNN disassembly by 60-Hz light entrainment with expected PNN loss (A) Experimental timeline for the 60 Hz light entrainment. V1, primary visual cortex. (B) Wisteria floribunda agglutinin (WFA)-labeled coronal brain sections of mice treated for 5 days with light control (no flickering, left) or 60 Hz (right). HP, hippocampus. Scale bar, 200 μm. Zoom-in, 50 μm. (C) Percentage of change in mean PNN-coated cells ± SEM in V1. Triangle, females. Circles, males. Two-sample t test. ∗∗∗p < 0.001. SEM, standard error of the mean.

This section outlines the light stimulation protocol for inducing PNN disassembly with 60 Hz light entrainment with the previously built device. An overview of the 60 Hz light stimulation protocol and the expected results can be found in Figure 4.

27. Acclimatize animals after they have arrived from the animal facility for 1 h.

***Note:*** To avoid potential impact of circadian rhythm on brain activity (Hor et al., 2019), we always stimulated the animals at the same time (in our case from 9 to 11 a.m.) and collected the tissue four hours after the last stimulation.

28. Remove all enrichment from the cage before stimulation to prevent the mouse from hiding.

***Note:*** The animals should always have unlimited access to food and water.

29. Position the animal cage in the marked position in the center of the box.

***Note:*** Up to 4 animals can be simultaneously stimulated.

30. Start the Arduino software as described in the previous section in steps 21–24 and select the stimulation protocol for either 60 Hz or control non-flickering light but with the same light intensity.
31. Expose the mice to light stimulation for 2 h per day for 5 consecutive days (Figure 4A).

***Note:*** During the stimulation the box lid was closed.

***Note:*** This test paradigm causes approximately 40% PNN reduction in V1 (Figures 4B and 4C). We have no insights into whether prolonged treatment will further reduce PNN and how fast the PNN recovers. According to the recovery rate after 3× KXA administration, the PNN recovers by 20% within 3 days after KXA withdrawal (Figure 2C).

***Note:*** We typically perfused the animal 4 hours after the last stimulation and performed image analysis. For detailed protocols see Venturino et al. (2021). Alternatively, this protocol could be explored for *in vivo* electrophysiological recordings in awake mice, behavioral studies, and *in vivo* imaging experiments to name a few.

***Note:*** The stimulation device generates electrical noise, therefore further modifications, such as isolating the electrical connections with copper mesh, are required to enable *in-vivo* electrophysiology during the 60 Hz light stimulation. Troubleshooting 4

## Expected outcomes

Both repeated ketamine anesthesia and 60 Hz light entrainment protocols led to a significant reduction in the number of PNN-coated cells (Figures 2B and 4C). KXA treatment is effective in V1 and S1. After 2×, 3×, and 6× treatment with KXA, we found a 50%, 80%, and 90% reduction in PNN-coated cells in the S1, respectively (Figure 2B). For the 60 Hz light entrainment, we found a 40% reduction in PNN-coated cells in the V1 (Figure 4C), which corresponds approximately to 2× KXA treatment.

Microglial cells in these cortical areas will contain PNN fragments within their lysosomes (Figures 6G and 6H). For further details refer to Venturino et al. (2021).

## Quantification and statistical analysis

Here, we describe first how to quantify the PNN density in a semi-automatic way, and second how to analyze the amount of PNN material within microglial lysosomes in 3D using Imaris software. The images are 40× z-stack confocal images immunostained for PNNs with WFA (*Wisteria floribunda* lectin), for microglia with Iba1 (ionized calcium-binding adaptor molecule 1), and for lysosomes/endosomes with CD68 antibodies. For further details about tissue preparation, histology, immunostaining, and confocal imaging settings see Venturino et al. (2021).

### PNN density quantification

**Timing: up to 30 min**

In the following example, we use data from the primary visual cortex (V1) of a female C57BL/6J mouse exposed to control light.

1. Software requirements:
  a. Get access to Imaris and obtain a license: https://imaris.oxinst.com/packages.
  b. Get access to MATLAB and obtain a license here: https://www.mathworks.com/products/get-matlab.html.
  c. Activate the MATLAB plugins for Imaris following this tutorial: https://imaris.oxinst.com/learning/view/article/enabling-imagej-fiji-and-matlab-plugins-in-imaris.
  d. To perform the described analysis, the following functions and plugins are required:
    i. the Imaris spot function,
    ii. the surface rendering functions, and the surface-surface coloc MATLAB plugin.
    iii. The MATLAB plugins for Imaris, called XTension, can be downloaded from the Imaris open repository at this link: https://imaris.oxinst.com/open/. Once the MATLAB plugins have been downloaded, add them to Imaris by selecting File > Preferences > Custom Tools.

***Note:*** The folder with the plugins can be linked by using the “Add” button.

The folder that includes the .exe and .xml files must contain the file ImarisLib.jar.

***Note:*** If this file is not present, you can copy the .jar file included with Imaris. This file is available in the path where Imaris was installed on drive C: C:\ProgramFiles\Bitplane\Imarisx64 9.3.1\XT\rtmatlab\ImarisLib.jar. At the end of this process, restart Imaris and the plugins will appear in the Image Processing dropdown menu.

2. Convert the confocal image file to an .ims file using the Imaris converter software.

***Note:*** Make sure that during the conversion the voxel size dimensions are not lost. Troubleshooting 5

**Figure 5:**
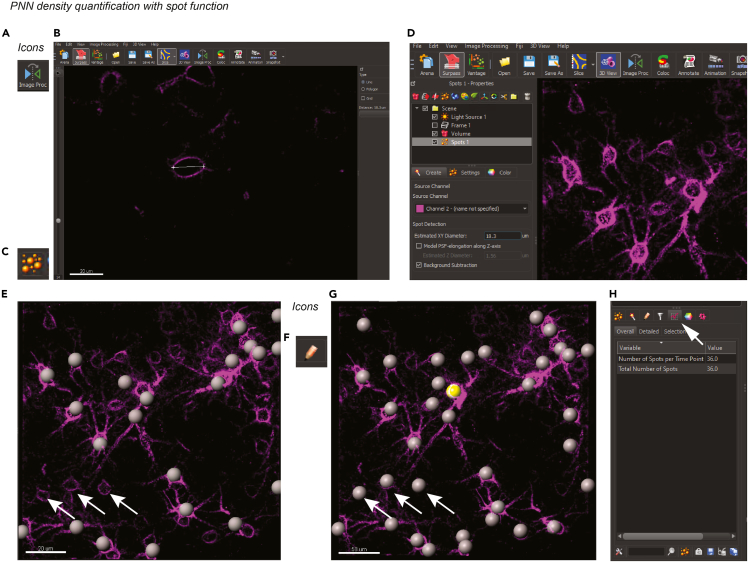
PNN density quantification protocol with IMARIS spot function Screenshots of Imaris software user-interface for spot function. (A) Imaris icon for image processing. (B) Measurement of PNN diameter of Wisteria floribunda agglutinin (WFA)-labeled cell in *Slice* mode. Scale bar, 20 μm. (C) Imaris icon for spot function. (D) Setting the spot detection parameters. (E–G) The spot function automatically detects spots indicated by grey balls. Scale bars: 20 and 50 μm, respectively. Arrows, PNN-coated cells, where the software failed to automatically detect. (F) Imaris icon for edit mode. Those spots are added manually (G). (H) The number of detected spots can be exported with the statistic function. Arrow, Imaris icon for statistics.
5. Visualize the PNN channel. Troubleshooting 6
6. Measure the diameter of PNNs in slice-visualization mode (Figure 5B). The length of the line is in the top right corner. Use the left navigation bar to go through the z stack.

***Note:*** Use the mouse wheel to zoom in.

3. Open the converted file in Imaris.
4. Apply filters by clicking the Image Processing icon (Figure 5A) on the toolbar and selecting *Median 3×3×3* and *Background Subtraction* for each individual channel.

7. Add a spot function by clicking on the spot icon (Figure 5C) and entering the diameter measured in step 6 (Figure 5D).

***Note:*** We recommend to use spot function instead of surfaces to count the absolute number of PNN because the surfaces can be fragmented during the rendering process, which would be counted as a single surface and artificially increase the numbers.

***Note:*** We refrain from measuring the PNN intensity signal for quantifying changes in density because this method is prone to artifacts which can be introduced by fluctuation of the laser power during the images acquisition, bleaching of the fluorophores during the scanning, different staining quality between brain slices, etc. (Waters, 2009).

***Note:*** An alternative to the spot function can be the quantification of the area or volume occupied by the PNN, which can be done in ImageJ/Fiji without additional plugins needed. The 3D quantification of the PNN phagocytosed by microglia, based on Imaris surface function, will be discussed in the following step-by-step method paragraph.

8. Click “Next” to visualize the automatically-detected spots (Figure 5E). By changing the threshold of the histogram in the lower left corner, new spots can be automatically added.

***Note:*** Some PNNs will not be detected. They can be manually added, as described next.

9. Click “Next” to end the detection.
10. Go to the edit mode by clicking on the pencil icon (Figure 5F).
11. Manually add the missed spots by positioning the mouse cursor on the missed PNNs, and pressing *Shift + left mouse click* (Figure 5G). Remove misplaced spots by pressing *Shift + left mouse click* on the spot.
12. Read the number of the detected spots in the statistic function (Figure 5H). In this example, 36 spots were detected.
13. Calculate the PNN density as $d=\frac{\text{n }\left(\text{PNN}\right)}{\text{V z}-\text{stack}}\;\text{where V z}-\text{stack}=\text{x∗y∗z}$ in spreadsheet software (for example Excel) and report the result as the number of PNN-coated cells per mm^3^.

***Note:*** Spot counting is also possible with open-source programs. The number of PNN-coated cells can be calculated using the spot counter plugin (https://github.com/nicost/spotCounter/) in ImageJ/Fiji (https://fiji.sc/). Another possibility is the Spot Detection Utilities plug in (https://gitlab.pasteur.fr/bia/spot-detection-utilities) for the software Icy (http://icy.bioimageanalysis.org/), which allows spot counting and 3D-tracking.

### Quantification of the amount of PNN material within microglial lysosomes

**Timing: up to 30 min**

In the following example, we will use data from the primary somatosensory cortex (S1) of a male C57BL/6J mouse treated with 2× KXA.

***Note:*** In our experiments, we use the lysosomal/endosomal marker CD68 for the quantification of the PNN material inside microglia. Other lysosomal markers are available such as Lamp1, Lamp2a, and Lamp2b. It is also possible to omit the lysosomal marker and focus on PNN material within microglia.

14. Convert the confocal image file to an .ims file using the Imaris converter software.

***Note:*** Ensure that during the conversion the voxel size dimensions are not lost.

**Figure 6:**
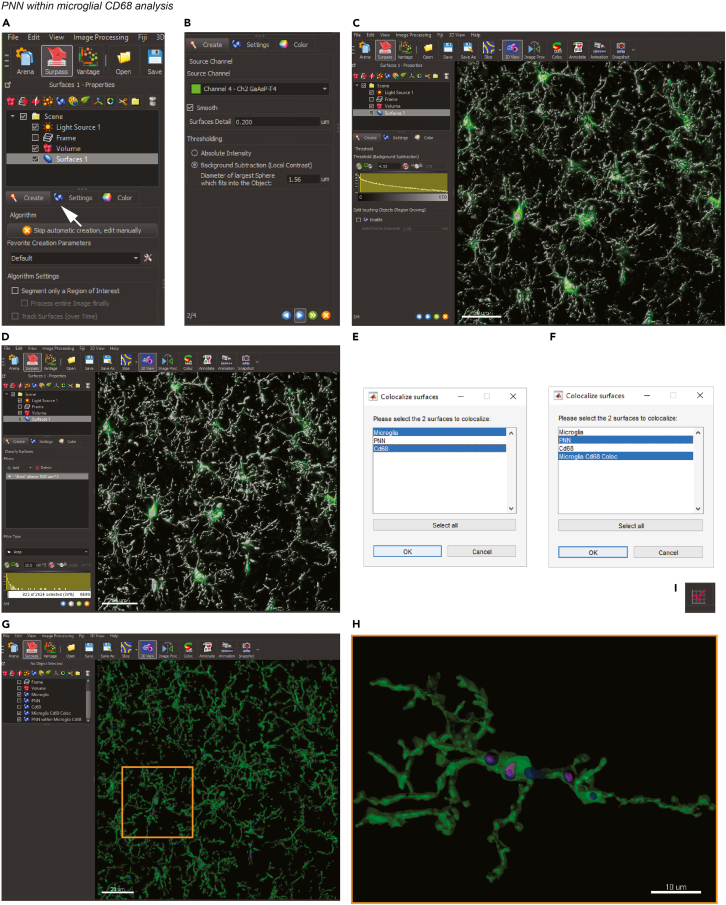
Determine PNN fragments within microglial CD68 with IMARIS surface rendering Screenshots of Imaris software user-interface for surface rendering. (A) Creating a surface module for Iba1-stained microglia (green). Arrow, Imaris icon for surface function. (B) Setting of the microglia surface parameter. (C) Microglia surface rendering with local contrast. Scale bar, 20 μm. (D) Application of the *Area* filter to remove rendering artifacts. (E and F) Apply *Surface-surface coloc* plugin for surface. (G and H) (G) Example image of colocalized surfaces for microglia(green), PNN (magenta), and CD68 (blue) with zoom-in (H). Scale bar, 20 μm. Zoom-in, 10 μm. (I) Imaris icon for statistics.
18. Click “Next”, select the microglia channel in the drop-down menu and enter the surface detail resolution. In our analysis, we used a surface detail of 0.2 μm. Select “Background subtraction (local contrast)” as the thresholding method (Figure 6B) and click “Next”.

***Note:*** The “surface detail” changes as a function of the resolution of the confocal image.

***Note:*** Using the local contrast as the thresholding method prevents the creation of artifacts due to fluctuations in the staining intensity. Troubleshooting 7

15. Load the file into Imaris.
16. If necessary, apply filters such as median filter, background subtraction, or similar using the Image Processing function (Figure 5A).
17. Activate the microglia channel and create a new surface by clicking on the surface icon (Figure 6A).

19. Cover the microglia volume with the surface by moving the threshold histogram toward the left side (Figure 6C) and click “Next”.

***Note:*** The little surface artifact that might occur will be filtered out in the next step.

***Note:*** The same values of thresholding can be used when the images are acquired at the same day, the laser power was stable, and the staining quality being comparable within the brain slice. Note: If the analysis is done with Imaris, we recommend to select the *local contrast* instead of the *intensity* for thresholding during the surface rendering process, to prevent artifact formation, which would result in overestimation of the 3D surfaces.

20. Correct for the surface artifacts by applying a filter. In the filter type drop-down menu select “Area” and apply a filter of 10 μm^2^ (Figure 6D). Click “Next” to end the surface creation process.
21. Repeat steps 17–20 for the PNN and the CD68 channels.
22. Rename the surfaces as *Microglia*, *PNN* and *CD68* by double clicking on them.
23. Perform “Resample 3D” to speed up the computation. Click on *Edit > Resample 3D* and enter 50 as the new size of the z axis. Click “OK”.

***Note:*** “Resample 3D” reduces the voxel density in a data set to increase the computation speed.

***Note:*** Resampling reduces the number of voxels in a grid but keeps the original relationship between the voxels.

24. Select the microglia and CD68 surfaces and click on *Image Processing > Surfaces Functions > Surface-surface Coloc* to open the MATLAB plug in.
25. In the MATLAB window, select the surfaces to colocalize (Figure 6E) and click “OK”.
26. In the next pop-up window, click on “Smoothing”.
27. Enter the smoothing factor. The smoothing factor must be double the image voxel size (in our case: 0.4 μm). Click “OK”.
28. At the end of the computation, a new surface will be created. Rename it as *Microglia Cd68 Coloc.*
29. Select the *PNN* and the *Microglia Cd68 Coloc* surfaces and repeat the colocalization process as described in steps 24–27 (Figure 6F).
30. At the end of the computation, a new surface will be created. Rename it as *PNN within Microglia CD68.*
31. Uncheck the Volume module and verify the presence of colocalized surfaces (Figures 6G and 6H).
32. Export the statistics file from all the created surfaces by clicking on the statistics icon (Figure 6I). Save the file in *.*xls or .csv format.
33. In a spreadsheet software (e.g., Excel), calculate the percentage of microglial volume occupied by CD68 and the percentage of the CD68 volume within microglia occupied by PNN.

***Note:*** The created *surface area* or *volume* can be exported as an Imaris statistics file (see point 19) and used; for example, to estimate the PNN covered surface after the treatments.

## Limitations

The repeated ketamine treatment is less invasive than other PNN-removal strategies such as injecting chondroitinase into the brain (Pizzorusso et al., 2002; Romberg et al., 2013; Carulli et al., 2016). On the other hand, the ketamine dosage is still high, making the translation to clinical practice challenging. Lower, repeated ketamine dosage at dissociative concentration is commonly used to obtain a schizophrenic-like rat model (Kaushik et al., 2020). These data suggest that the effects of ketamine on the brain might be dose-dependent and its actual effect is less obvious. Apart the well-described inhibition of the NMDA receptors (Autry et al., 2011), ketamine seems to have a plethora of different target sites, from sodium channels to cholinergic, dopamine, and opioid receptors (Wray and Rasenick, 2019) (Sleigh et al., 2014): this makes characterization of the molecular and cellular pathways involved challenging.

60 Hz light entrainment successfully mimics the gamma oscillations induced by ketamine (Ahnaou et al., 2017; Akeju et al., 2016). With *in-vivo* electrophysiological recordings in awake, freely-moving mice, we observed strong 60 Hz oscillations evoked in the V1 (Venturino et al., 2021), suggesting that the visual cortex is tuned to the stimulation frequency. How far 60 Hz oscillations can spread in the brain is still not clear. 40 Hz light-induced oscillations have also been recorded in the hippocampus (Iaccarino et al., 2016) and in the prefrontal cortex (Jones et al., 2019), suggesting that light-induced oscillation can also reach other cortical and subcortical regions.

A limitation of the quantification side of this protocol is that we performed our analysis on a commercial software provided by the Bioimaging facility of IST Austria. Imaris combines an intuitive, user-friendly software interface to represent and analyze 3D images. Custom-made scripts from MatLab or python can be implemented as plugins in Imaris XTension.

Alternative strategies to visualize and analyze 3D images and surfaces provides VAA3D (Peng et al., 2010) (https://alleninstitute.org/what-we-do/brain-science/research/products-tools/vaa3d/) and the python-based Napari (https://napari.org/tutorials/fundamentals/installation.html#installing-as-a-bundled-app), which is based on an open source plugin (https://www.napari-hub.org/).

## Troubleshooting

### Problem 1

The KXA solution is not clear (step 1).

### Potential solution

Prepare a fresh KXA solution for every experiment. If the precipitates are visible, filter the solution using 0.2 μm syringe filters.

### Problem 2

The mouse does not become anesthetized and reflexes are still present (step 5–6).

### Potential solution

Make sure that the correct dosage of KXA was administered and that the injection was performed *i.p.* If you can see an accumulation of liquid under the skin, then the solution was not injected in the peritoneal cavity. In this case, exclude the mouse from the study.

### Problem 3

The Arduino code doesn’t work (step 22).

### Potential solution

There are several reasons why the code might not work. Here are possible reasons: first, the parenthesis is open but not closed; or second, the semicolon is missing at the end of the command line.

In case, the error message says “COM Port not found”, navigate in the Arduino IDE to *Tools > Port* and make sure that the COM Port is checked.

### Problem 4

The 60 Hz light stimulation device generates electrical noise during *in-vivo* electrophysiology recordings (step 31).

### Potential solution

To perform *in-vivo* electrophysiology during the light stimulation, it is necessary to electrically isolate every solder connection, building a small Faraday cage around each. To do this, wrap a small piece of copper shielding mesh around the connection and fix it with electrical tape. Isolate the transistor in the same way.

### Problem 5

The voxel dimensions are lost during the conversion process (step 2 in the “quantification and statistical analysis”).

### Potential solution

Check the voxel dimension in the metadata file of the confocal file and enter the correct voxel sizes in Imaris by pressing *CTRL + I*.

### Problem 6

The channel adjustments tool is not visible, the channels cannot be activated or deactivated (step 5 in the “quantification and statistical analysis”).

### Potential solution

Navigate to *Edit > Show Display Adjustment* or click *CTRL + D*.

### Problem 7

The surface rendering with the local contrast thresholding method is not precise because the staining signal is low (step 18 in the “quantification and statistical analysis”).

### Potential solution

If you have a low signal-to-noise ratio from the staining, we recommend to select *Brightness* as the thresholding method during the Imaris surface rendering process, although this method is less precise in signal detection.

## Resource availability

### Lead contact

Further information and requests for resources and reagents should be directed to and will be fulfilled by the lead contact, Dr. Sandra Siegert (sandra.siegert@ist.ac.at).

### Materials availability

This study did not generate new unique reagents.

## Data Availability

This study did not generate any unique datasets or codes.
